# Metastatic Gastric Mucosal Melanoma: A Rare Case Presenting With Diffuse Gastric Polyposis

**DOI:** 10.7759/cureus.43740

**Published:** 2023-08-19

**Authors:** Zhi Hao Yan, Jingxiang Huang, Jianbang Chiang, Kah Wai Clarence Kwan

**Affiliations:** 1 Department of Gastroenterology and Hepatology, Sengkang General Hospital, Singapore, SGP; 2 Department of Pathology, Sengkang General Hospital, Singapore, SGP; 3 Division of Medical Oncology, National Cancer Centre Singapore, Singapore, SGP

**Keywords:** braf-v600e mutation, immunotherapy, unknown primary, metastatic, gastric polyps, mucosal melanoma

## Abstract

We report a 66-year-old Chinese lady who presented with a three-month history of postprandial vomiting, early satiety, anorexia and weight loss, and significant physical findings of hepatomegaly and ascites. Gastroscopy revealed gastric polyposis with both hyperpigmented and unpigmented lesions over the gastric fundus, body, and proximal antrum, biopsies of which yielded malignant melanoma histologically. Cross-sectional imaging with CT also demonstrated extensive hepatic and bony metastases. No cutaneous or ocular primary was detected. She was treated with a combination of ipilimumab and nivolumab but developed interval progression of hepatic metastases after two cycles of immunotherapy. The patient eventually succumbed two months after diagnosis.

## Introduction

Melanomas are epithelial cancers originating from melanocytes, which are present most abundantly in the skin. When found within the gastrointestinal tract, melanomas are most often metastatic from a primary cutaneous lesion [1], although they can also arise de novo within the gut mucosal epithelium. Making the distinction between primary and secondary melanomas can be difficult in the absence of a cutaneous lesion [2]. While more than 85% of primary gastrointestinal mucosa melanomas occur in the anorectal and oropharyngeal regions [3], metastatic melanomas of the gastrointestinal tract most commonly affect the small bowel (51-71%), followed by the stomach (27%), colon (22%) and esophagus (5%) [4]. Here, we report a case of metastatic gastric mucosal melanoma with an unusual endoscopic appearance of diffuse gastric polyposis.

## Case presentation

A 66-year-old Chinese lady presented to the emergency department with a three-month history of postprandial vomiting, early satiety, anorexia and unintentional weight loss, preceded by a one-year history of nausea, non-vertigo giddiness, and a persistent sense of disequilibrium. She had co-morbidities of hypertension and dyslipidemia and a history of colonic polyps, which were detected at colonoscopy one year ago during evaluation for constipation. The patient also reported a family history of breast cancer in her mother and lung cancer in her brother. Physical examination was remarkable for the presence of hepatomegaly, ascites, and bipedal edema. She was otherwise anicteric and did not exhibit peripheral stigmata of chronic liver disease. Cardiopulmonary, neurological, and breast examination yielded no abnormalities, and no cervical, axillary, or inguinal lymphadenopathy was elicited.

**Figure 1:**
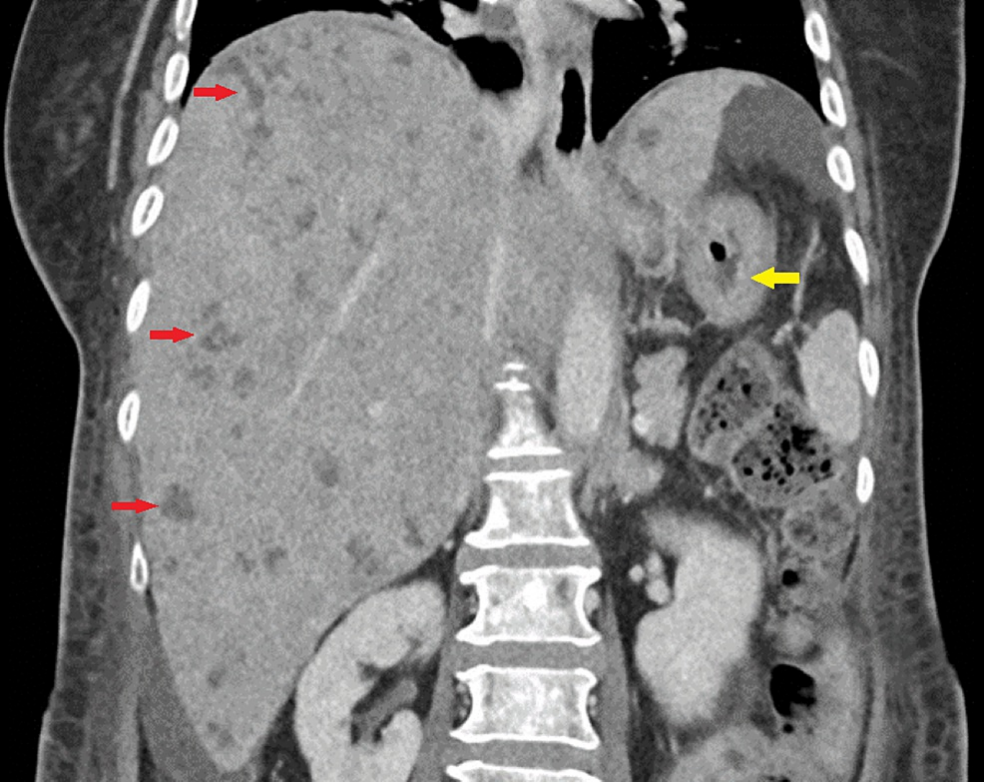
CT of the abdomen showing multiple hypodense hepatic lesions (denoted by red arrows) and mural thickening with mucosal hyperenhancement in the stomach (denoted by yellow arrow)

At presentation, she had a normal haemoglobin level of 12.8 g/dL, reduced serum albumin level of 27 g/L, and a predominantly cholestatic pattern of liver function derangements with elevated alkaline phosphatase at 460 U/L and gamma-glutamyl transferase level of 338 U/L, but normal total bilirubin level of 18 µmol/L and alanine and aspartate transaminase levels of 33 U/L and 47 U/L, respectively. Computed tomography (CT) of the abdomen and pelvis (Figure 1) revealed a grossly enlarged liver with innumerable scattered hypodense lesions, marked mural edema and mucosal hyperenhancement in the proximal stomach, and multiple lytic and sclerotic foci in the vertebrae.

**Figure 2:**
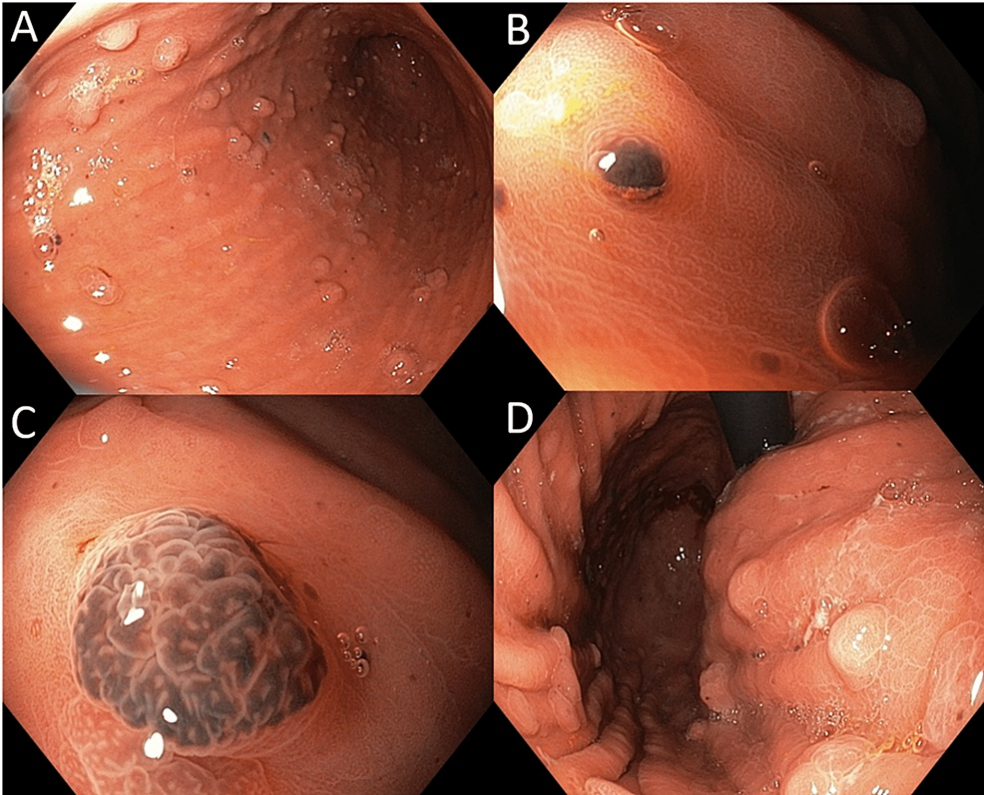
OGD findings (A) Multiple gastric polyps in the body and proximal antrum, with scattered punctate pigmented spots. (B) Diminutive melanotic polyp in the gastric body. (C) Magnified view of the melanotic polyp. (D) Multiple gastric polyps in proximal stomach and fundus, viewed in retroflexed position. OGD: oesophagogastroduodenoscopy

Oesophagogastroduodenoscopy (OGD) (Figure 2) revealed approximately 50 sessile polyps ranging from 0.1 to 0.5 cm in diameter diffusely distributed across the gastric fundus, body, and proximal antrum. The majority of the polyps were unpigmented and macroscopically appeared to be benign polyps while some were distinctly hyperpigmented. None of the polyps appeared to be a dominant lesion. Punctate, flat, pigmented spots were also scattered across the gastric mucosa. No ulcerative lesions were otherwise observed in the stomach, and the esophagus and duodenum were endoscopically normal. Representative biopsies were obtained separately from the hyperpigmented and unpigmented polyps.

**Figure 3:**
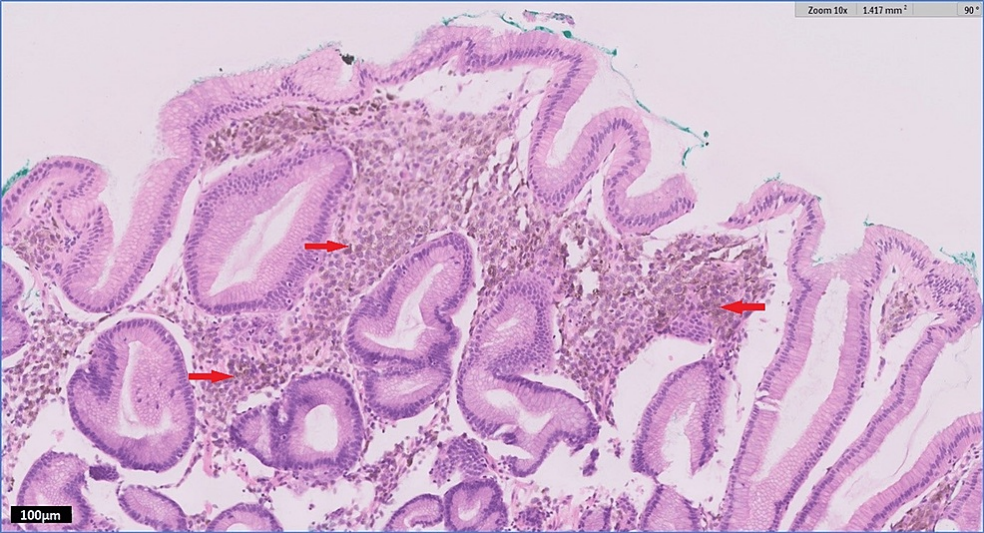
H&E-stained section at 100x magnification showing atypical melanocytic lesion composed of aggregates of pigmented cells (denoted by arrows) in the superficial lamina propria, between or around benign pits

**Figure 4:**
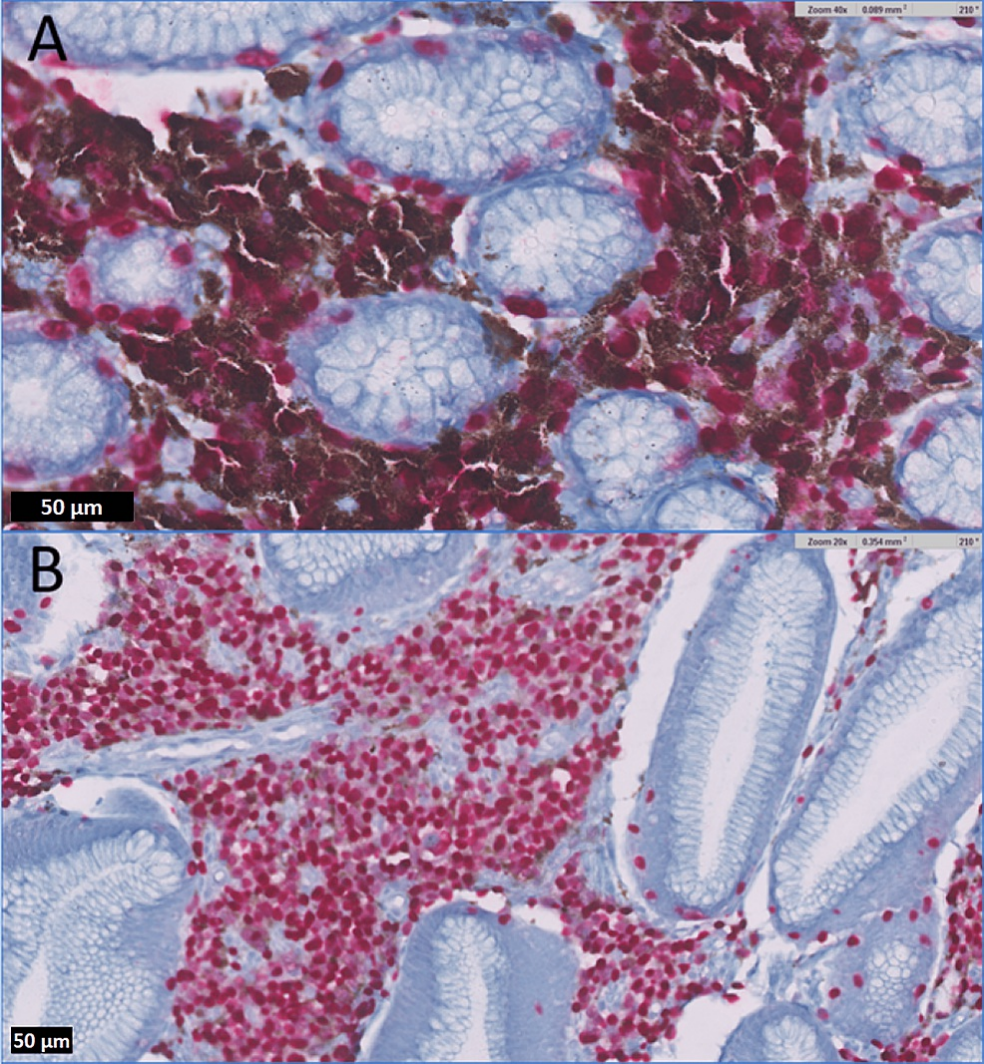
(A) SOX-10 immunostain at 400x magnification showing SOX-10 positive lesional cells in a hyperpigmented area. (B) SOX-10 immunostain at 200x magnification showing SOX-10 positive lesional cells in a hypopigmented area.

Histopathological examination (Figure 3) of tissue from the hyperpigmented lesions demonstrated irregular aggregates of atypical melanocytes between or around benign pits, with nuclear atypia. Interestingly, tissue from the unpigmented lesions also yielded rounded aggregates of atypical melanocytes within the superficial lamina propria and harbored a higher proliferation index, assessed by expression of Ki-67, than the heavily pigmented lesions. Both the hyperpigmented and unpigmented lesions exhibited positive immunohistochemical staining for SOX-10 (Figure 4), Melan-A, and faintly, HMB-45. Overall, these findings were confirmatory of malignant melanoma. Molecular analysis by targeted next-generation sequencing revealed a BRAF V600E mutation.

Colonoscopy was unremarkable apart from a solitary 6 mm sigmoid colon tubular adenoma, which was excised by cold snare polypectomy. No 18-F-fluorodeoxyglucose (FDG)-avid lesion was identified on positron emission tomography-computed tomography (PET-CT) to suggest a primary lesion or any other metastatic lesion along the gastrointestinal tract.

Head-to-toe skin examination was conducted by a dermatologist, with findings of three seborrhoeic keratoses on her thighs and a solitary skin-colored lump with a central punctum on the left upper back, suggestive of an epidermal cyst. None of the cutaneous lesions were deemed suspicious. Notably, the patient reported that she had a hyperpigmented skin lesion on her left face excised by her general practitioner four years earlier, which returned histopathological findings of an epidermal cyst and an adjacent small intradermal naevus with no increased mitotic activity or cytologic atypia. The ophthalmological and anogenital examination did not yield any significant findings. Staging CTs of the brain and chest also returned negative.

In light of the high visceral metastatic disease burden, a decision was made to initiate treatment within one week of diagnosis, albeit with palliative intent. The patient was commenced on doublet immunotherapy comprising the immune checkpoint inhibitors nivolumab at 3 mg/kg every three weeks and ipilimumab at 1 mg/kg every three weeks. A peritoneal drainage catheter was inserted for severe symptomatic ascites. Unfortunately, she continued to deteriorate in performance status and developed progressively worsening hyperbilirubinemia and liver function tests. Persistently high ascitic drainage output was also complicated by depletion hyponatremia. Repeat abdominal CT after two cycles of immunotherapy demonstrated interval worsening of hepatic metastases. Severe thrombocytopenia also ensued, with a nadir platelet count of 7 x 10^9^/L at five weeks from initiation of immunotherapy, from her pre-treatment count of 173 x 10^9^/L. A diagnosis of immune checkpoint inhibitor-related thrombocytopenia was made in consultation with a hematologist after competing diagnoses were excluded. In light of disease progression, deterioration in performance status, and the development of an immune-related adverse event, immunotherapy was halted and she was placed on best supportive care. Our patient eventually passed away two months from the time of melanoma diagnosis.

## Discussion

Gastric mucosal melanomas, whether primary or metastatic, are typically described to be ulcerative or mass-like lesions [3,5-11]. To our knowledge, this is the first reported case of metastatic gastric mucosal melanoma presenting with gastric polyposis of mixed melanotic and amelanotic morphology, in a patient who did not otherwise have a history of long-term proton pump inhibitor use or known familial polyposis syndromes. The amelanotic polyps could have masqueraded as benign polyps, if not for the telltale presence of synchronous melanotic lesions and high clinical index of suspicion for malignancy in view of known hepatic metastases. The literature search yielded one other report of multifocal metastatic gastrointestinal melanoma presenting with multiple gastrointestinal polyps [12], but in this case, there were numerically far fewer gastric polyps - five in total in the stomach, which all appeared pigmented macroscopically, and extragastric involvement including an ulcerated polyp in the second part of the duodenum and five pigmented polyps in the colon.

Our case also highlighted the challenge in distinguishing between primary and secondary gastric mucosal melanoma, in the absence of a cutaneous primary. We regarded the disease as metastatic with unknown or regressed cutaneous primary, in view of the multifocality of gastric involvement with no clear dominant lesion, and the presence of the BRAF V600E oncogene mutation, which is more common in (though not exclusive to) cutaneous melanomas as compared to primary mucosal melanomas [13,14]. The absence of extragastric (in particular, small bowel) involvement was also conspicuous in our patient, as melanomas have been known to exhibit a predilection for metastasis to the small bowel. This is postulated to be due to strong melanoma cell surface expression of chemokine receptor 9 (CCR9), which in turn promotes migration of tumor cells to the small bowel where chemokine ligand 25 (CCL25), the ligand of CCR9, is strongly expressed [15]. We acknowledge that the absence of FDG-avid bowel lesions on PET-CT did not definitively exclude small bowel involvement, but we could not justify performing video capsule endoscopy in our patient, as there was no further clinical indication, and extensive evaluation for other metastatic lesions would not have altered clinical management significantly.

Metastatic mucosal melanoma portends a poor prognosis, in spite of immunotherapy that has revolutionized the treatment of cutaneous melanomas with the possibility of long-term disease control [16]. Interestingly, our patient had a BRAF V600E driving mutation that is rarely seen in mucosal melanoma and may have responded to treatment with a combination BRAF and MEK inhibitor. Our patient had a long symptomatic period prior to presentation and a high metastatic disease burden by the time of diagnosis and, unfortunately, experienced disease progression despite combination immune checkpoint inhibitor therapy. Her rapid clinical deterioration and decline in performance status precluded further lines of systemic therapy.

Asians tend to have a higher incidence of acral and mucosal melanoma, making up nearly half of all melanomas seen [17-19]. Ideally, we would have liked to perform germline genetic testing in this patient with multiple unpigmented and hyperpigmented sessile gastric polyps and a strong family history of cancer, to look for pathogenic variants in CDKN2A and other familial polyposis genes. This would have informed management for her family if an underlying pathogenic variant was found. However, the patient was too ill to undergo pre-test genetic counseling, and hence, testing could not be performed.

## Conclusions

In conclusion, we present an unusual case of metastatic gastric mucosal melanoma with a unique endoscopic appearance of diffuse gastric polyposis comprising both melanotic and amelanotic lesions. Our case underscores the importance of comprehensive clinical evaluation, endoscopic and histopathological examination, and molecular analysis to accurately diagnose and manage malignant melanoma of the gastrointestinal tract. While immunotherapy has shown promising results in the treatment of advanced melanoma, there remains a need for continued research and exploration of novel therapeutic approaches to improve outcomes for patients with advanced and aggressive disease.
